# A population-based study of large granular lymphocyte leukemia

**DOI:** 10.1038/bcj.2016.59

**Published:** 2016-08-05

**Authors:** M V Shah, C C Hook, T G Call, R S Go

**Affiliations:** 1 Division of Hematology, Mayo clinic, Rochester, MN, USA; 2 Robert D. and Patricia E. Kern Center for the Science of Health Care Delivery, Mayo Clinic, Rochester, MN, USA

## Abstract

Large granular lymphocyte (LGL) leukemia is a lymphoproliferative disorder of cytotoxic cells. T-cell LGL (T-LGL) leukemia is characterized by accumulation of cytotoxic T cells in blood and infiltration of the bone marrow, liver or spleen. Population-based studies have not been reported in LGL leukemia. We present clinical characteristics, natural history and risk factors for poor survival in patients with LGL leukemia using the Surveillance, Epidemiology, and End Results Program (SEER) and the United States National Cancer Data Base (NCDB). LGL leukemia is an extremely rare disease with the incidence of 0.2 cases per 1 000 000 individuals. The median age at diagnosis was 66.5 years with females likely to be diagnosed at 3 years earlier compared with males. Analysis of patient-level data using NCDB (*n*=978) showed that 45% patients with T-LGL leukemia required some form of systemic treatment at the time of diagnosis. T-LGL leukemia patients have reduced survival compared with general population, with a median overall survival of 9 years. Multivariate analysis showed that age >60 years at the time of diagnosis and the presence of significant comorbidities were independent predictors of poor survival.

## Introduction

Large granular lymphocyte (LGL) leukemia is a chronic lymphoproliferative disorder of cytotoxic T or natural killer cells. T-cell LGL (T-LGL) leukemia is characterized by clonal proliferation of CD3^+^/CD8^+^ cytotoxic T lymphocytes and infiltration of the bone marrow, liver or spleen. Clinically, T-LGL leukemia may be present as asymptomatic lymphocytosis or may be associated with autoimmune diseases, such as rheumatoid arthritis or cytopenias. The diagnosis of T-LGL leukemia is based on peripheral blood expansion of the leukemic LGL population (>0.5 × 10^9^/l) in association with CD3^+^/CD8^+^ phenotype and evidence of clonality as suggested by clonal rearrangement of the T-cell receptor gene using PCR or flow cytometry.^1, 2, 3^

T-LGL leukemia is a rare disease representing ~2–5% of chronic lymphoproliferative diseases in the US.^3^ Despite significant advances in understanding biology and therapeutics of LGL leukemia, the incidence, demographics, natural history, treatments and long-term prognosis of the disease remain poorly characterized.

Here, we present the first population-based study of T-LGL leukemia studying epidemiology and demographics using the Surveillance, Epidemiology and End Results (SEER) Program and the National Cancer Data base (NCDB). We discuss long-term outcomes for patients with T-LGL leukemia compared with matched general population and factors predicting adverse outcomes.

## Materials and methods

SEER is a program of the National Cancer Institute (NCI) that collects and publishes cancer incidence and survival data covering ~28% of the US population. The SEER*Stat software (version 8.1.2; NCI, Bethesda, MD, USA) was used to obtain de-identified individual-level and survival data from the SEER-18 registries.^4^ NCDB, a joint program of the Commission on Cancer and the American Cancer Society, is a nationwide oncology outcomes database for >1500 cancer programs in the US and Puerto Rico capturing about 70% of all newly diagnosed cases of cancer in the US Patient-level data were obtained from the NCDB Participant User File and analyzed for demographic patterns as well as overall survival (OS).^5^

The SEER and NCDB databases were queried using ICD-O histology code 9831 corresponding to LGL leukemia. Incidence rates (cases/1 000 000) were calculated using 2000–2011 data from SEER and age-adjusted to the US standard population for the year 2000. Relative survival was calculated using SEER*Stat analysis. Briefly, relative survival was defined as the ratio of the proportion of observed survivors in a cohort of LGL leukemia patients to the proportion of expected survivors in a comparable set of individuals that do not have LGL leukemia adjusting for the general survival of the US population for race, sex, age and time when the diagnosis was established.^4^ Patient-level data including age, gender, race, year of diagnosis, stage of the disease, treatment and survival were calculated using NCDB data. For NCDB data, T-LGL leukemia patients were identified using ‘grade' by restricting the analysis to grade 5 (T cell). Methotrexate and cyclosporine therapies were classified as chemotherapy, cyclosporine A was classified as immunotherapy and prednisone was classified as hormone therapy per NCDB methodology. Comorbid disease burden was calculated using Deyo adaptation (1992) of Charlson's comorbidity index (CCI). The score was mapped from as many as ten reported International Classification of Diseases, Ninth Revision, Clinical Modification (ICD-9-CM) secondary diagnosis codes.^6^

Statistical analyses were performed using JMP 10.0.1 (SAS Institute Inc., Cary, NC, USA). Kaplan–Meier analysis was used for survival analysis and the log-rank test was used to compare survival curves. Cox proportional hazards model was used to assess the influence of various prognostic factors on OS.

## Results

**Figure 1:**
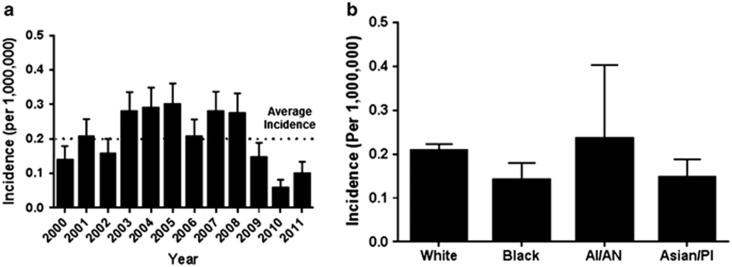
(**a**) The incidence of T-LGL leukemia by year of diagnosis. Each bar represents the mean incidence with error bars representing the s.e.m. The dotted line denotes average incidence over the period study; (**b**) The incidence of T-LGL leukemia by race during years 2000–2012 (AI/AN, American Indian or Alaskan Native; Asian/PI, Asian or Pacific Islander). The bars represent incidence per million individuals with error bars representing s.e.m.

The incidence of LGL leukemia was 0.2 cases per 1 000 000 individuals (range 0.17–0.23). The incidence did not change significantly between years 2000 and 2011 (Figure 1a). The incidences according to racial groups were (incidence, 95% confidence interval (CI)): White 0.2 (0.18–0.24), Black 0.14 (0.07–0.24), American Indian/Alaska Native 0.24 (0.03–0.8) and Asian/Pacific Islander 0.15 (0.07–0.25). The incidences among Blacks, American Indians/Alaska Natives or Asian/Pacific Islanders were not significantly different compared with Whites (Figure 1b).

Using the NCDB database, patient-level data was available for 1150 patients with LGL leukemia. Of these, 978 patients (85%) were diagnosed with T-LGL leukemia. The median age at diagnosis was 66.5 years (range, 19–90). A total of 14% patients were <50 years of age at the time of diagnosis. The incidence of T-LGL leukemia was similar between the sexes with a male-to-female ratio of 1.05. Females were diagnosed at a younger age compared with males (65 vs 68 years, *P*<0.05).

**Figure 2:**
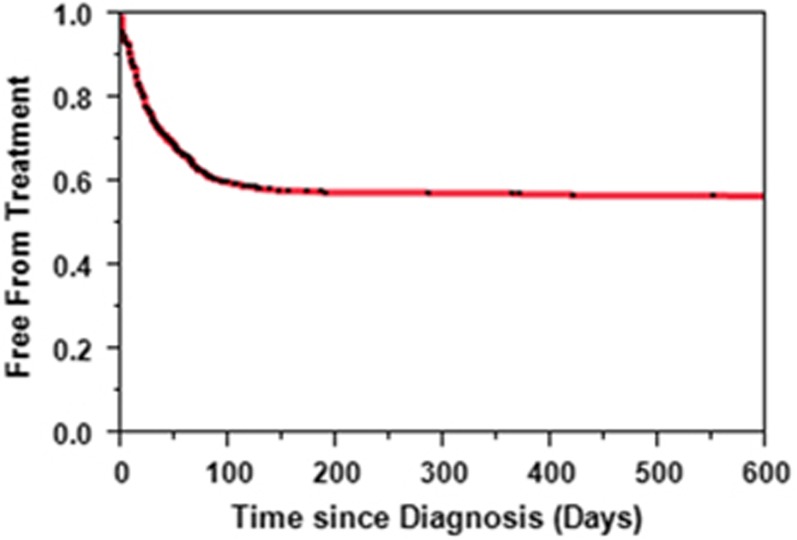
Treatment-free survival in patients with T-LGL leukemia.

In the cohort studied, 45% patients required systemic therapy. The median time to treatment was not reached (Figure 2). Among the patients who were treated for T-LGL leukemia, 60% patients were treated within 1 month of diagnosis, 37% were treated between 1–6 months of diagnosis, whereas data was not available for 3% patients. Treatments directed at T-LGL leukemia were described as chemotherapy, immunotherapy or hormone therapy. Chemotherapy was offered to 373 patients and was described as single-agent (*n*=328, 88% of treated patients) or multi-agent (*n*=27, 7%), whereas regimen was unknown in the rest. Fifteen patients refused chemotherapy that was offered. Thirty-five percent of patients received chemotherapy, 5% patients received immunotherapy and 13% patients received hormone therapy. A total of 22% patients received combination treatment with chemotherapy and hormone therapies, whereas 3% patients received chemo and immunotherapy. Ten (2.3%) patients received combination of hormone and immunotherapy whereas only 0.68% patients received the combination of chemo, immuno and hormone therapies. The OS was not different between those who received therapy and those who did not (data not shown).

**Figure 3:**
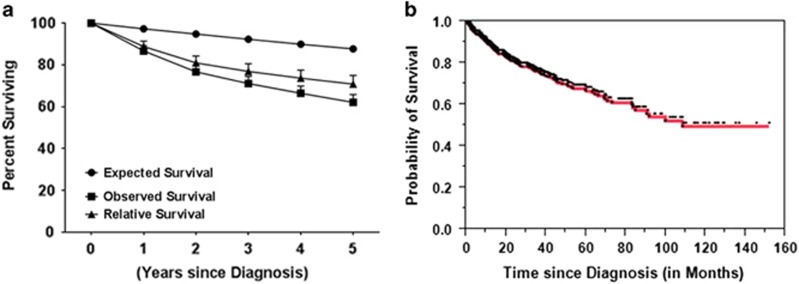
(**a**) Expected (•), observed (▪) and T-LGL leukemia-specific relative (▴) survival (mean±s.e.m.); (**b**) OS in patients with T-LGL leukemia. Each tick mark denotes censored event.

In the general US population, the expected survival for 12, 24, 36, 48 and 60 months is 97.30, 94.80, 92.30, 89.90 and 87.70%, respectively. In contrast, survival for patients with LGL leukemia at the same time points was 86.70, 76.70, 71.10, 66.40 and 62.10% (Figure 3a). With a median follow-up of 32 months (range 0–152), the vital status of 760 (77.7%) patients was known available for analysis. At the time of last follow-up, 75.5% patients were alive with median OS of 108.4 months (Figure 3b).

**Figure 4:**
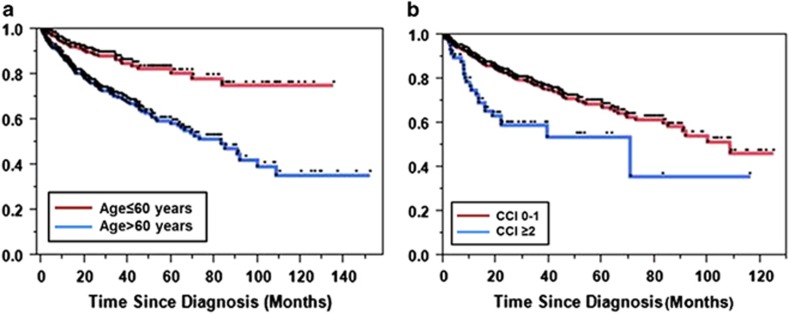
(**a**) OS in patients diagnosed either age >60 years (blue) or younger (red) at the time of diagnosis is a poor risk factor for patients with T-LGL leukemia; (**b**) OS in the presence (blue) or absence (red) of significant comorbidities defined as CCI >2 at the time of diagnosis in patients with T-LGL leukemia.

A multivariate analysis that included age at the time of T-LGL leukemia diagnosis, sex, race, year of diagnosis and CCI showed that age >60 years at the time of diagnosis, and higher comorbidity score (⩾2) were independent predictors of poorer survival (Table 1). Kaplan–Meier survival analysis showed the median OS for patients younger than 60 years or less at the time of diagnosis was not reached compared with median OS of 83 months for those older than 60 years at diagnosis (*P*<0.001, Figure 4a). Similarly, median OS for patients who carried significant comorbidities, as defined by CCI of ⩾2, fared poorly compared with patients with CCI of 0 or 1 (median OS 70.4 vs 108.4 months, *P*=0.0003, Figure 4b).

## Discussion

LGL leukemia is believed to be a rare disease characterized by clonal expansion of cytotoxic lymphocytes—either cytotoxic T lymphocytes or natural killer cells. Over the years, there has been a significant progress in understanding the biology of LGL leukemia. Most reports are either case reports or small series limiting our understanding of the impact of LGL leukemia in the population. Here, we present the first population study of LGL leukemia studying demographics, natural history and risk factors for poor survival using the SEER and the United States NCDB databases.

Our study confirms that LGL leukemia is an extremely rare disease with the annual incidence of 0.2 cases per million individuals in the US with no significant change in the annual incidence of LGL leukemia during the time of study. LGL leukemia is thought to be a disease of the elderly, though prior studies reported median age at the time of diagnosis varying anywhere between 39 and 67 years.^7, 8, 9, 10^ In our cohort of 978 patients with T-LGL leukemia, the median age of diagnosis was 66.5 years. Overall incidence was similar between males and females, though females were likely to be diagnosed 3 years earlier compared with males. We found 14% patients to be under the age of 50 years at the time of diagnosis, in contrast to previously described French registry database that reported 26% patients <50 years of age.^7^

A wide range (33–80%) of patients are reported to undergo therapy for T-LGL leukemia.^7, 8, 9, 10^ The challenge in part is due to the fact that the definition of systemic therapy for T-LGL leukemia can vary widely. Moreover, many patients receive steroids and/or immunosuppressive therapies for autoimmune diseases diagnosed before, concurrently or after the diagnosis of T-LGL leukemia—making it difficult to interpret the data.^3, 11^ In our cohort, less than half patients with T-LGL leukemia required some form of systemic treatment at the time of diagnosis. This pattern is significantly different compared with the published French registry data,^7^ though reasons behind it remain unclear.

The long-term outlook and risk for poor prognosis in patients with T-LGL leukemia are not well understood. T-LGL leukemia is considered to be a chronic disease with unclear impact on survival. In an observational study 17% patients died during the 2-year follow-up.^1^ In contrast, another study showed median survival of 10 years.^8^ Our data is consistent with the latter study with a median OS of 9 years. At all the time points studied, the observed survival in patients with T-LGL leukemia was shorter than expected survival suggesting that T-LGL leukemia adversely affects OS (Figure 3a).

In a single-institution retrospective study of 286 patients with T-LGL leukemia, anemia, severe neutropenia and lymphopenia were identified as poor prognostic factors.^12^ Although we did not have access to similar laboratory parameters, we found that diagnosis of LGL leukemia in the older patients (aged >60 years) and those with high coexistent comorbidities as defined by CCI>2 were independent predictors of poor survival.

The limitation of this study includes inability to access individual patient data (including laboratory parameters), thus limiting our understanding of the presenting symptoms, laboratory parameters and reasons to institute therapy. NCDB data does not include cause of death, limiting our ability to ascertain cause specific mortality. The SEER data presented here suggest that at all the time points studied, T-LGL leukemia adversely affects OS.

In summary, LGL leukemia is a rare disease that most commonly affects the elderly population. Fewer than half the patients with T-LGL leukemia required some form of systemic treatment at the time of diagnosis. Although OS of patients with LGL leukemia is 9 years, it shortens the survival compared with that of the general population. Age ⩾60 years at the time of diagnosis and the presence of significant comorbidities are independent predictors of poor survival in patients with T-LGL leukemia. Further studies are required to understand poor prognosis and to devise better treatments.

## Figures and Tables

**Table 1:** Multivariable analysis showing predictors of mortality in patients with T-LGL leukemia

*Parameters*	*Hazard ratio*	*Lower CI*	*Upper CI*	P*-value*
Age ⩽60 vs >60 years	2.73	1.90	4.04	<0.0001
Male vs female	0.82	0.61	1.09	0.19
White vs other	0.99	0.97	1.00	0.23
Charlson–Deyo score ⩽2 vs>2	2.03	1.29	3.06	0.003
Year of diagnosis (2001–2006 vs 2007–2012)	1.11	0.76	1.69	0.58

Abbreviations: lower CI, lower limit of confidence interval; upper CI, upper limit of confidence interval.
